# Evaluation of the Physical Stability of Starch-Based Hydrogels Produced by High-Pressure Processing (HPP)

**DOI:** 10.3390/gels8030152

**Published:** 2022-03-01

**Authors:** Dominique Larrea-Wachtendorff, Vittoria Del Grosso, Giovanna Ferrari

**Affiliations:** 1 Department of Industrial Engineering, University of Salerno, 84084 Fisciano, Italy; dlarrea-wachtendorff@unisa.it (D.L.-W.); vdelgrosso@unisa.it (V.D.G.); 2 ProdAl Scarl, c/o University of Salerno, 84084 Fisciano, Italy

**Keywords:** starch-based, high-pressure processing, stability

## Abstract

Starch-based hydrogels are natural polymeric structures with high potential interest for food, cosmeceutical, and pharmaceutical applications. In this study, the physical stability of starch-based hydrogels produced via high-pressure processing (HPP) was evaluated using conventional and accelerated methods. For this purpose, conventional stability measurements, namely swelling power, water activity, texture, and organoleptic properties, as well as microbiological analysis of rice, corn, wheat, and tapioca starch hydrogels, were determined at different time intervals during storage at 20 °C. Additionally, to assess the stability of these structures, accelerated tests based on temperature sweep tests and oscillatory rheological measurements, as well as temperature cycling tests, were performed. The experimental results demonstrated that the physical stability of starch-based HPP hydrogels was interdependently affected by the microorganisms’ action and starch retrogradation, leading to both organoleptic and texture modifications with marked reductions in swelling stability and firmness. It was concluded that tapioca starch hydrogels showed the lowest stability upon storage due to higher incidence of microbial spoilage. Accelerated tests allowed the good stability of HPP hydrogels to be predicted, evidencing good network strength and the ability to withstand temperature changes. Modifications of the rheological properties of corn, rice, and wheat hydrogels were only observed above 39 °C and at stress values 3 to 10 times higher than those necessary to modify commercial hydrogels. Moreover, structural changes to hydrogels after cycling tests were similar to those observed after 90 days of conventional storage. Data obtained in this work can be utilized to design specific storage conditions and product improvements. Moreover, the accelerated methods used in this study provided useful information, allowing the physical stability of starch-based hydrogels to be predicted.

## 1. Introduction

Hydrogels are a commercially widespread group of polymeric materials, consisting of three-dimensional crosslinked networks of hydrophilic or hydrophobic biopolymers capable of absorbing and retaining a significant amount of water [1]. In recent years, hydrogels have been recognized as “smart structures” with tailor-made characteristics conferring different functional attributes of the utmost importance for the design, synthesis, and self-assembly of novel biomaterials, including drug delivery systems [2,3]. Currently, natural hydrogels produced from renewable sources, as alternatives used to replace or reduce the use of synthetic materials, are receiving significant attention in the scientific community due to their compatibility with the human body [4]. Among this trending group of hydrogels, starch-based hydrogels are among the most promising alternatives for producing polymeric biomaterials [5,6]. Their biocompatibility, hydrophilicity, and biodegradability have been highlighted as remarkable characteristics of these structures, encouraging their extensive use in several applications [2,3,5,7,8,9,10,11]. Moreover, given the wide range of applications of starch-based hydrogels, their physical–mechanical properties and durability can be fine-tuned by changing the processing methods and operation conditions, the type of biopolymer, and the composition of the liquid phase [4].

Recently, high-pressure processing technology (HPP) was proposed to produce natural-starch-based hydrogels [12,13,14]. HPP is a non-thermal technology that is widely used in the food industry for pasteurization, since it allows extended shelf life, minimizing nutritional and sensorial property losses in processed products [15]. Moreover, it is well known that HPP promotes sol–gel transitions in proteins and other food components [16]. For this reason, this technology has also been proposed as a physical method for modifying or gelatinizing different types of starch suspensions, allowing the limitations of gelation processes that are currently utilized to be overcome, such as the long operation times, high energy consumption levels, and use of hazardous materials [13,17,18,19,20,21,22,23,24,25].

Starch-based hydrogels form thanks to the physical entanglement of polymer chains or other non-covalent interactions, giving a reversible nature to these structures, which represents an advantage in biomedical and food applications for these materials. However, due to their non-permanent bonds, HPP hydrogels are commonly considered weak structures with reduced rheological properties compared to chemical hydrogels. This hinders their utilization in applications where good mechanical properties and stability are desired [4]. It is interesting to note that starch-based HPP hydrogels obtained in well-identified processing conditions display excellent mechanical and rheological properties that are even superior to those of thermal gels, making them very promising materials [12,13].

Nevertheless, to the best of our knowledge, no studies on the physical stability of these novel structures have been carried out, the determination of which is of the utmost importance in view of improving the design of these biomaterials, optimizing the processing conditions, and proposing their application in different industrial sectors.

This work aimed at evaluating the stability and physicomechanical characteristics of starch-based HPP hydrogels with time by applying different stress conditions, using either specific conventional or accelerated detection methods, and determining the shelf life of hydrogels over short storage periods. Bearing in mind that the guidelines for stability testing (International Conference on Harmonization, ICH, [26]) commonly used to predict the physical stability of cosmeceutical and pharmaceutical products under different ambient conditions (temperature, relative humidity) utilize long sampling periods even in accelerated conditions (from 3 to 6 months), are expensive and time-consuming, and require considerable scientific expertise and knowledge of the materials and methodologies involved [26], we attempted to develop specific testing methods to determine the stability of the new materials. These methods, which are less expensive and time-consuming, allow reproducible and high-quality data correlated with extreme environmental conditions to be obtained that are useful for innovative product development.

## 2. Results and Discussion

### 2.1. Conventional Evaluations

#### 2.1.1. Microbiological Analysis

**Figure 1:**
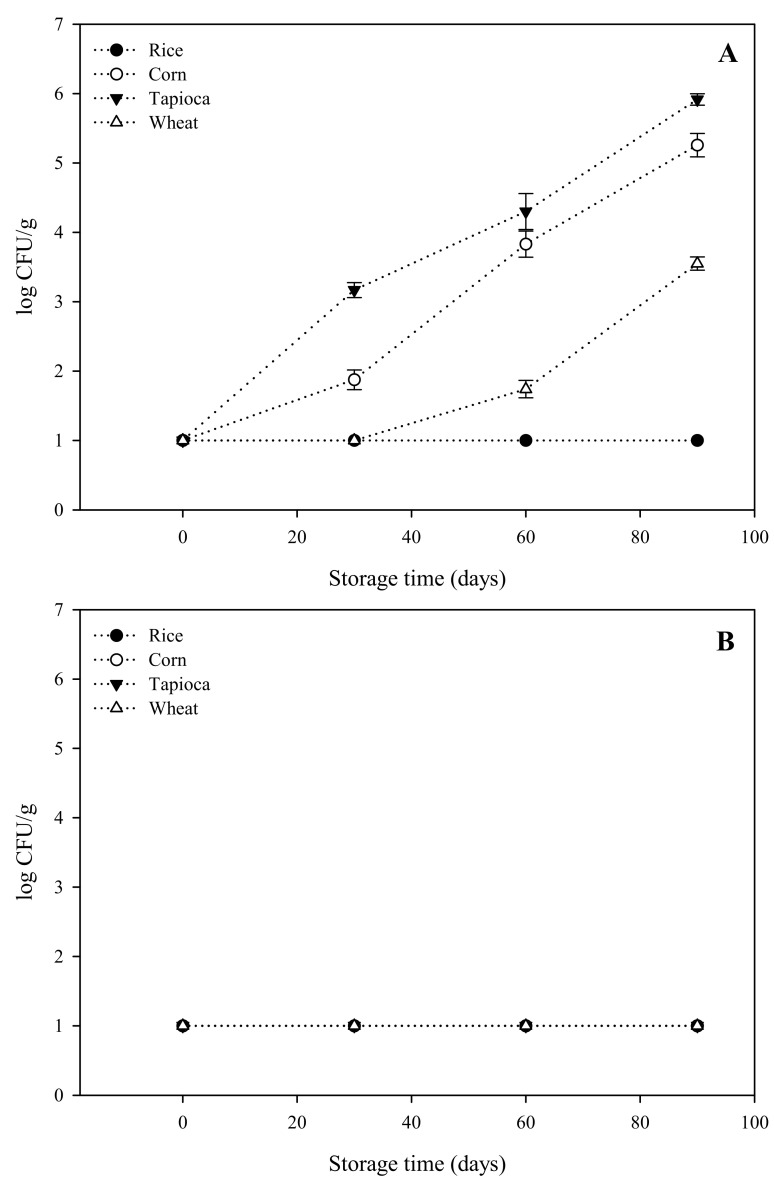
Growth curves of aerobic mesophilic total count (**A**) and yeasts and molds (**B**) of starch-based HPP hydrogels during the storage period. Symbols are means of three measurements ± standard deviations (S.D).

Growth curves of aerobic mesophilic microorganisms, yeasts, and molds on starch-based HPP hydrogels stored at 20 °C are shown in Figure 1.

The initial microbial counts in all starch-based hydrogels tested were within the undetectable range, namely < 1 log CFU/g (Figure 1). Moreover, no yeasts or molds were detected, and the number of survivors was always below the detection limit (<1.0 log CFU/g) during the entire storage period (Figure 1B), confirming the effectiveness of HPP to inactivate this group of microorganisms. It has been extensively demonstrated that yeasts and molds are very sensitive to HPP due to the instantaneous impact of pressure over the nuclear membrane of yeast and mold populations, which causes a lethal injury to microbial cells, leading to their complete inactivation [27,28]. In contrast, although immediately after HPP treatments the microbial load was below the detection limit, significant growth of aerobic mesophilic microorganisms in almost all starch-based hydrogels was detected during storage (Figure 1A). While the microbial load of rice HPP hydrogels remained below detection limits (<1.0 log CFU/g) during the entire storage period, in tapioca, corn, and wheat hydrogels we detected exponential growth of aerobic mesophilic bacteria from the first month of storage onwards. After 90 days of storage, the microbial counts of the three hydrogels were above 6, 5, and 3 log CFU/g, respectively. These results confirmed the well-known findings that HPP treatments cause sublethal injuries on certain resistant microorganisms that are able to repair cellular damages due to pressure and recover their viability during storage, as reported elsewhere [28,29]. In this regard, it can be hypothesized that the resistant survivors detected in corn, wheat, and tapioca-starch-based HPP hydrogels could belong to pressure-resistant microbial subpopulations such as bacterial spores [30], which seemingly differ based on the type of starch source and should be addressed in further experiments. Bacterial spores are extremely resistant life forms triggered by stress scenarios such as high-pressure conditions, returning to active growth during storage at optimal temperature conditions such as 20 °C [30,31]. However, further experiments on the identification and individual aspects of the pathogenicity of the microbial populations in starch-based hydrogels should be performed.

Furthermore, the results obtained gave interesting insights into the microbiological fingerprint of starch-based hydrogels produced by HPP, indicating that microbiological stabilization of these materials should be a matter of concern during processing.

#### 2.1.2. Swelling Stability

**Figure 2:**
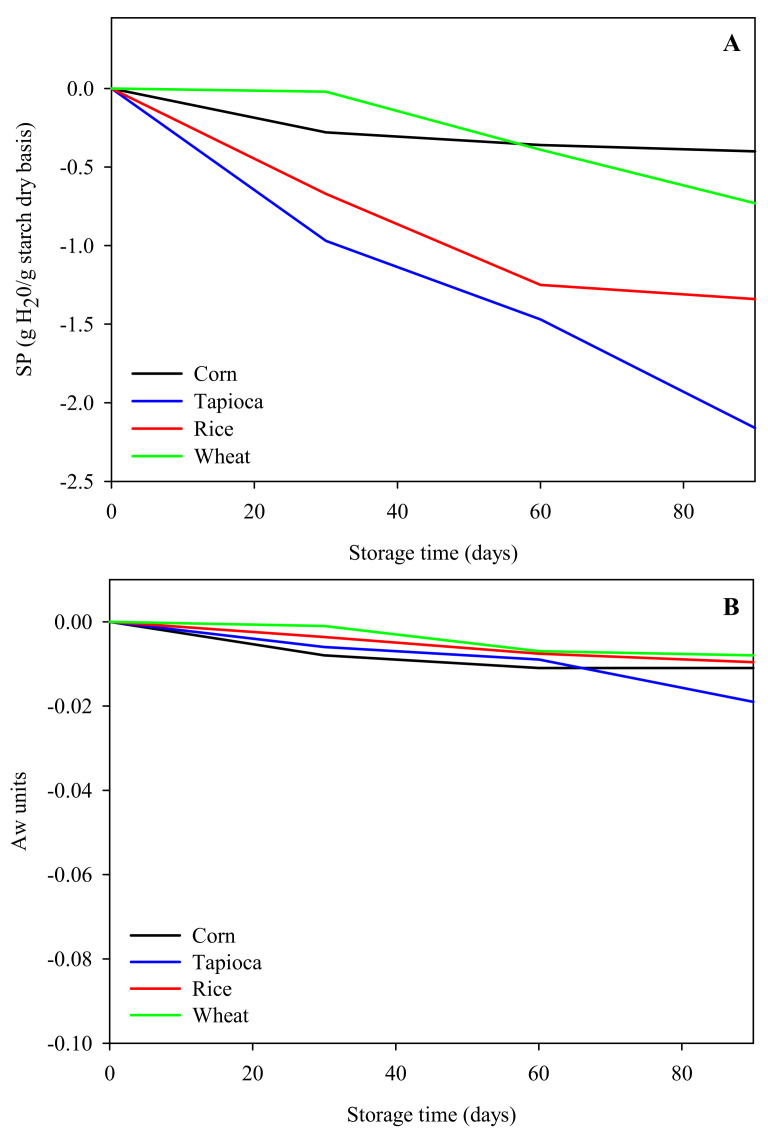
Influence of storage time on the swelling power (**A**) and A_w_ (**B**) values of starch-based HPP hydrogels.

Water is the main solvent that influences the processing, quality, texture properties, and stability of starch-based hydrogels [23,32,33,34]. The ability of starches to bind water during gel formation and retain it during storage can be assessed by measuring parameters such as the swelling power (SP) and water activity (A_w_). Figure 2 depicts the evolution (delta of current and initial values) of SP and A_w_ values of corn, rice, wheat, and tapioca HPP hydrogels during storage at 20 °C.

At the beginning of the storage period (0 days), starch-based HPP hydrogels presented similar A_w_ values (in the range of 0.974–0.969). Tapioca starch hydrogels showed the highest initial SP values (7.4 g H_2_O/g _dry starch_), followed by rice (6.5 g H_2_O/g _dry starch_), corn (5.4 g H_2_O/g _dry starch_), and wheat starch hydrogels (5.16 g H_2_O/g _dry starch_). These results can be explained by the higher capacity of tapioca starch granules to swell and solubilize with respect to cereal starch granules and the absence of amylose–lipid complex formation, as reported elsewhere [14,22,35].

Nonetheless, an evident syneresis phenomenon was observed, as shown in Figure 2A, where for all samples the SP values decreased with increasing the storage time. Wheat and corn starch hydrogels displayed better swelling stability than rice and tapioca starch hydrogels. For the latter structures, an abrupt reduction of water binding capacity from the beginning of the storage period was detected and losses at the end of the storage period (90 days) of 1.3 g H_2_O/g _dry starch_ for rice and −2.2 g H_2_O/g _dry starch_ for tapioca starch hydrogel were measured. These results are in slight agreement with the ones reported by Torres et al. [35] for potato starch gels obtained by thermal treatments utilizing different types of water. The authors observed a syneresis phenomenon after 60 days of cold storage (4 °C) in samples prepared with distilled water, at potato starch concentrations of 10% and 20% *w/w*. However, it should be emphasized that either the gel formation conditions (60 °C for 30 min) or the storage temperature (4 °C) was more severe, and could have delayed the physical and microbiological deterioration of the hydrogels. In our case, it could be assumed that the differences observed among samples can be attributed to the different starch retrogradation extent in hydrogel samples. Retrogradation has been defined as a structural reorganization of the starch chains due to the crystallization of amylose and amylopectin molecules during storage, causing gel shrinking and phase separation (syneresis) [36]. The rate and extent of retrogradation are sensitive to the water contents of starch gels and baked products, because water acts as a plasticizer of the amorphous region [37,38]. Therefore, it can be assumed that the starch retrogradation extent was higher in hydrogels with higher initial SP values, such as tapioca and rice starch hydrogels, than in hydrogels with lower initial SP values, such as corn and wheat starch hydrogels. Microbial spoilage may accelerate retrogradation in tapioca starch hydrogels, promoting higher syneresis, confirming the results of Figure 1A and Figure 2A.

Measurements of A_w_, which accounts for the status and dynamics of water in association or interaction with other molecules in complex matrices [39], were carried out. As shown in Figure 2B, in all samples a slight and constant decrease in A_w_ was observed during storage. Through A_w_ measurements, it was possible to evaluate the free water present on the surface of the sample, which changed only marginally with storage time. On the contrary, water loss, also quantified as free water in SP measurements, during storage time was much higher than that detected through A_w_ due to the centrifugal forces applied in this method. These discrepancies suggest that the water entrapped in the polymeric network experienced different changes upon storage, moving outwards layer by layer, reaching the hydrogel surface from where it was easily removed during SP measurements.

#### 2.1.3. Texture Profile Analysis (TPA)

**Figure 3:**
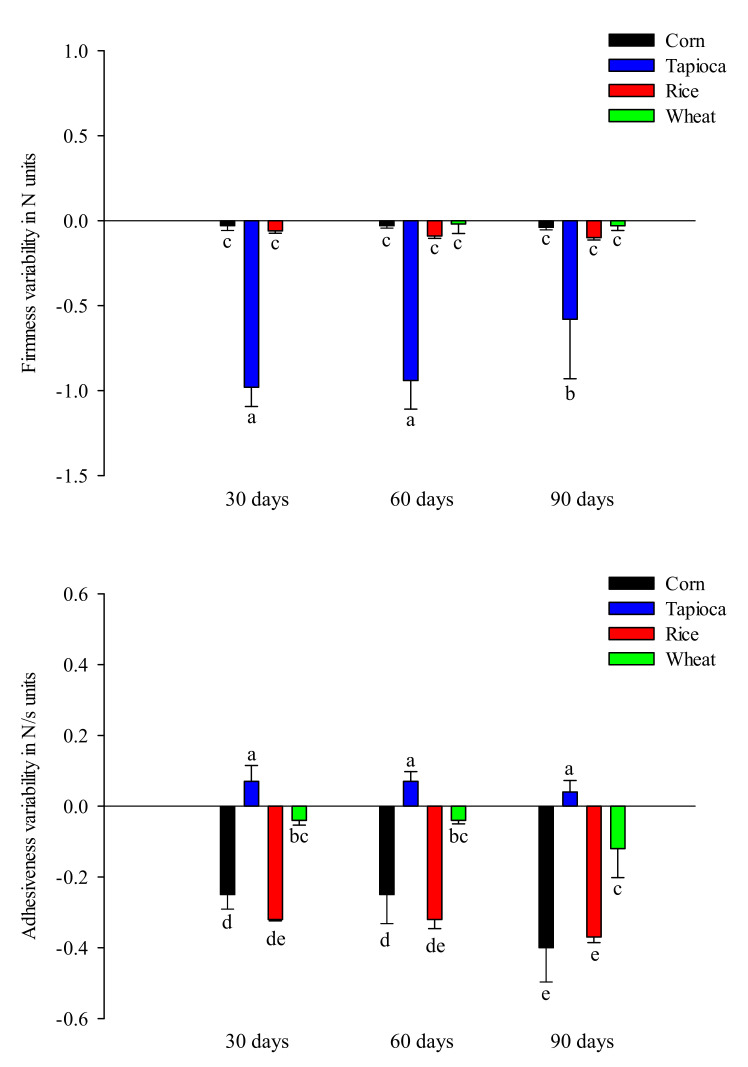
Influence of storage time on firmness and adhesiveness values of starch-based HPP hydrogels. Different letters above the bars indicate significant differences among the mean values (LSD, *p* ≤ 0.05).

The physical stability of polymeric materials is defined as the extent to which a product retains, within the specified limits, the same properties and physical characteristics possessed at the time of its packaging [26]. Texture measurements represent valuable tools to quantify the physical properties of polymeric materials such as starch-based hydrogels. Moreover, based on our previous findings [12], the firmness and adhesiveness are the parameters characterizing the texture of starch-based HPP hydrogels. Consequently, the trends of these two parameters were determined for rice, wheat, corn, and tapioca starch hydrogels during the storage period and the data are reported in Figure 3 as the delta of current and initial values.

The firmness gives information on the strength of a gel structure, whereas the adhesiveness accounts for the adhesive forces present in starch-based hydrogels [14]. As can be seen in Figure 3, the texture of starch-based HPP hydrogels was strongly affected by storage time. In particular, the firmness values of all hydrogel samples decreased throughout the storage period, with tapioca starch hydrogels showing the highest reductions in this parameter (>70%) after 30 days of storage at 20 °C. Rice, corn, and wheat starch hydrogels showed slight reductions in firmness during the entire storage period (90 days). These results agree with those previously discussed (Figure 1 and Figure 2), confirming that the physical stability of these structures is strongly influenced by factors triggered during the storage period, such as microbiological spoilage and starch retrogradation, which cause strong structural changes, such as network weakening, phase separation, and syneresis.

Similarly, as the storage time increased, the adhesiveness of rice, corn, and wheat starch hydrogels decreased due to the viscoelasticity losses of the gel network. Interestingly, the increased adhesiveness of tapioca starch hydrogels after 30 days of storage can be attributed to the leaching of water, amylose, and amylopectin, which determine adhesive forces in starch gels [40].

#### 2.1.4. Organoleptic Property Evaluation

**Figure 4:**
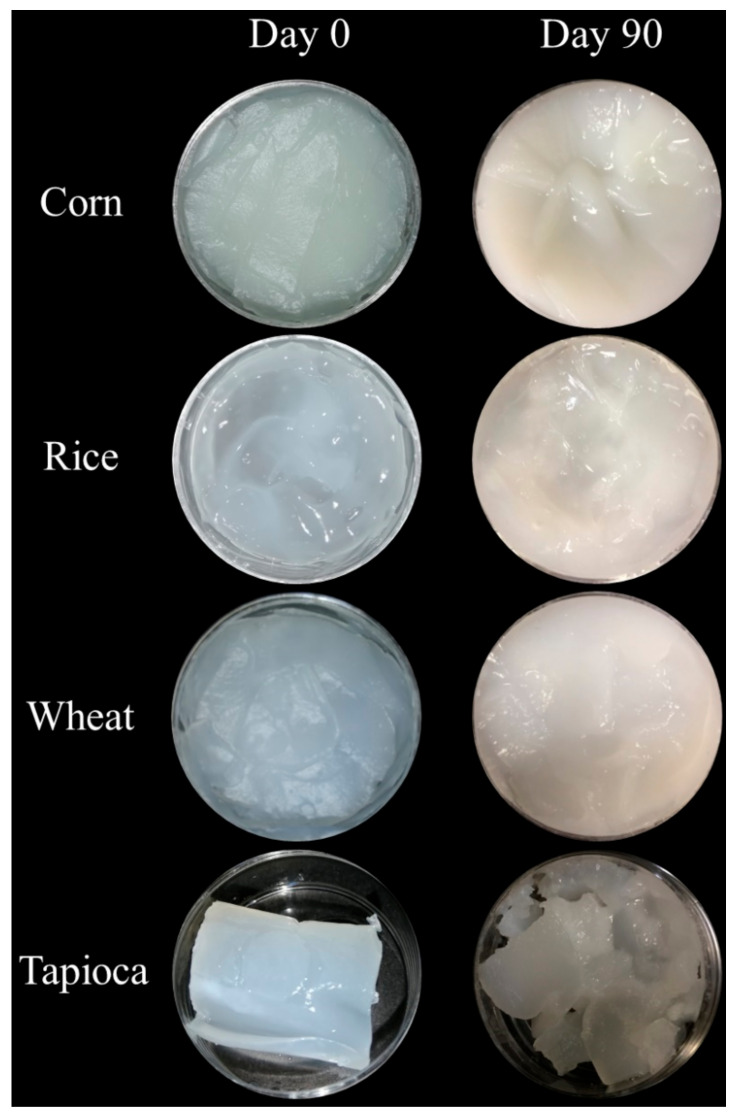
Influence of storage time on the physical appearance of starch-based HPP hydrogels.

Data for the organoleptic properties of rice, corn, tapioca, and wheat starch hydrogels measured during storage are reported in Table 1 and Figure 4.

In agreement with the results of the previous sections, tapioca starch hydrogels showed more significant organoleptic changes than rice, corn, and wheat hydrogels. Syneresis and phase separation after the first month of storage, and consequently, the rupture of the structure after 60 days, were detected via the organoleptic evaluation as the main physical changes of tapioca starch hydrogels during storage. Interestingly, only slight organoleptic modifications of rice starch hydrogels were observed during the storage period (Table 1, Figure 4). These findings suggest that the organoleptic changes in starch-based hydrogels are strongly influenced by the actions of microorganisms, which produce network deteriorations and facilitate starch retrogradation. Moreover, the level of the organoleptic changes depended on the structural characteristics of each hydrogel (Figure 4). For instance, in cream-like structures such as rice, corn, and wheat hydrogels, the organoleptic changes are less visible than in rubber-like structures such as tapioca starch hydrogels, highlighting the importance of instrumental determination in the evaluation of the physical stability of such gel structures.

Furthermore, from conventional stability results, it can be hypothesized that the stiffer the starch-based HPP hydrogel is, the more marked the structural deterioration, meaning weaker physical stability can be expected when microbial spoilage promotes starch retrogradation. Starch, on which the hydrogels under investigation in this work are based, is a polymer chain of glucose molecules linked to each other through glycosidic bonds that can undergo important modifications during storage. The structural reorganization of its macromolecules and the attack of spore fungiform microorganisms that normally possess amylases [41] can synergically produce the weakening of the internal structure of the starch hydrogels due to the rupture of the physical bonds or interactions, causing organoleptic changes, firmness reductions, syneresis, and phase separation.

### 2.2. Accelerated Stability Tests

In accelerated stability tests, a product is subjected to high-stress conditions. For the sake of completeness, the applied stress required to cause starch-based hydrogels structure failure was determined in this work. These data can be utilized to predict the capability of products to keep their initial characteristics, as well as for processing improvements [26]. In our experimental trials, parameters such as temperature and mechanical stress were set as stress conditions during accelerated stability testing, which are widely used to assess the stability of gels and pharmaceutical products [42,43].

#### 2.2.1. Temperature Cycling Test

Temperature cycling tests are accelerated physical methods that are commonly utilized in pharmaceutical sciences to provide information on a product’s instabilities that is not provided by isothermal tests [44]. Cyclic temperature tests are designed based on the characteristics of the products. In this study, starch-based HPP hydrogels were packaged and stored at 4 °C and 40 °C, and the temperature was changed every 24 h for 7 days, simulating extreme storage conditions [43]. The results of temperature cycling tests on the texture parameters of starch-based HPP hydrogels are reported in Table 2.

The data reported in Table 2 show that the textures of all hydrogels were significantly affected by the temperature cycling test conditions. Rice, corn, and wheat starch hydrogels showed significant reductions in firmness and adhesiveness values (*p* ≤ 0.05), whereas a sharp decrease in firmness was observed in tapioca starch hydrogels (−88%) (*p* < 0.05), as well as an increased adhesiveness (*p* ≤ 0.05) after the cycling tests. These results could be attributed to the leakage of water and solubilized amylose, or amylopectin molecules promoted by temperature changes (cycles from 4 °C to 40 °C), causing changes in the interactions occurring in the gel network and the adhesive forces. This is in agreement with the findings reported by Schirmer et al. [45]. The authors stated that viscosity changes can be detected in starch–water systems in the temperature range between 30 °C and 50 °C as a result of the solubilization (leaching) of macromolecules.

Furthermore, the results of the temperature cycling tests showed that physical changes in cream-like hydrogels are more related to the network weakening than to rupture, differently from what was observed in tapioca starch hydrogels. Remarkably, these physical changes are similar to those observed after the conventional storage period, highlighting the effectiveness and accuracy of this accelerated method to predict the physical instabilities of starch-based HPP hydrogels in shorter time periods.

#### 2.2.2. Rheology

Rheological tests have been proposed to predict the stability of products and provide useful information to improve formulations or overcome the occurrence of certain instabilities in gels structures [43]. For this purpose, temperature and stress sweep tests were carried out, which are valuable tools for predicting the physical stability of emulsions, gels, and pharmaceutical products [43,46,47].

##### Temperature Sweep Tests

**Figure 5:**
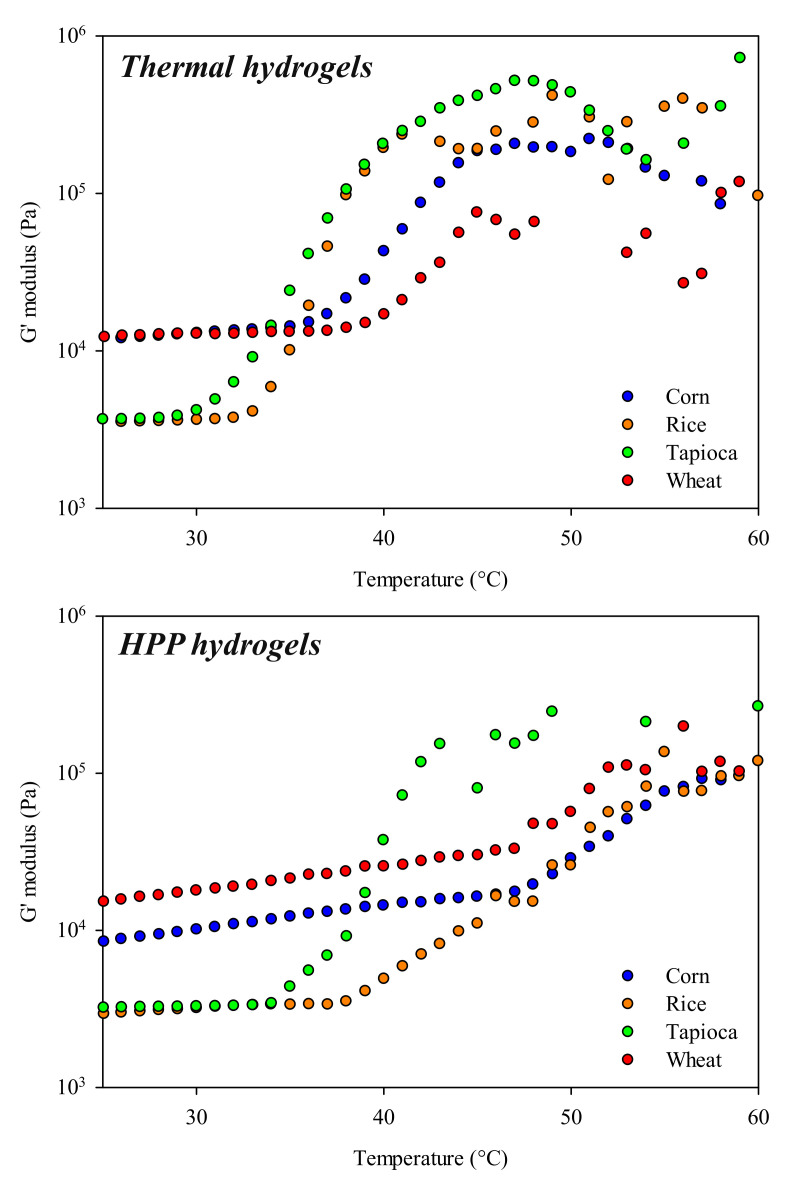
The influence of temperature stress on the elastic response (G′ modulus) of starch-based hydrogels immediately after HPP treatments and thermal treatments.

To predict the thermal stability of the starch-based HPP hydrogels studied in this investigation, temperature sweep tests from 25 °C to 60 °C at a heating rate of 1 °C/min were performed. For the sake of comparison, thermal gels were also tested. Figure 5 depicts the elastic responses (G′) of corn, rice, tapioca, and wheat hydrogels undergoing temperature stress.

From Figure 5, it can be observed that the temperature increments influenced the rheological properties of starch-based HPP hydrogels to different extents. Elastic instabilities occurred in corn and wheat HPP hydrogels over 47 °C, whereas the same instabilities were detected in rice and tapioca HPP hydrogels at 39 °C and 35 °C, respectively. Moreover, the inflection points of G′ curves, corresponding to the temperatures affecting the gel-like profiles of hydrogels, were always observed at higher temperature values (±6.5 °C average) in hydrogels produced using high-pressure processing than in those obtained with thermal processes, suggesting a superior thermal stability of the former ones.

It is well known that gels obtained with physical methods exhibit a strong dependence of the G′ modulus on temperature, with significant decreases in elastic domains observed with increasing temperature, mainly due to the loss of interconnectivity of the network constituents [43]. Interestingly, an opposite trend can be observed in Figure 5, with the elastic domains (G′) of starch-based hydrogels being wider and the inflection points moving towards higher temperature values. This can be attributed to the retrogradation phenomenon typically observed in starch gels produced with thermal treatments, and to a less extent in HPP-treated starch [48,49,50,51,52].

Moreover, some studies have demonstrated that differing from thermal gelatinization, in HPP starch gels a characteristic retrogradation phenomenon is likely to occur due to the different dynamics of water and the presence of almost intact starch granules (absence of stirring). This prevents lower amylose leaching, which in turn is less prone to retrograde [48,49,50]. This may also partially explain the higher thermostability of starch-based HPP hydrogels.

##### Stress Sweep Tests

**Figure 6:**
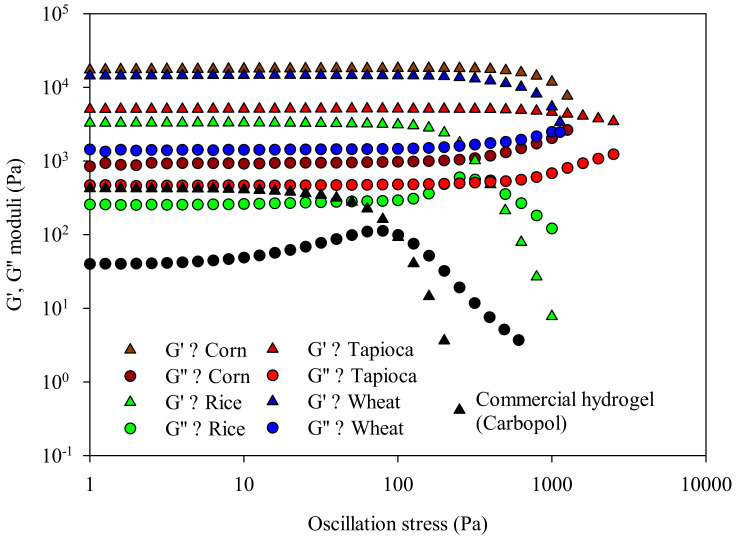
Influence of deformation stress on the viscoelastic responses (G′, G″ moduli) of starch-based hydrogels immediately after HPP treatments.

The network strength of starch-based HPP hydrogels, and for the sake of comparison of a commercial hydrogel, namely Carbopol, was evaluated through stress sweep tests. In Figure 6, the data of the viscoelastic responses (G′ elastic and G″ viscous moduli) of corn, rice, tapioca, wheat hydrogels, and Carbopol subjected to a deformation (oscillation) stress ramp are reported.

All samples showed the typical behavior of gels structures, with elastic properties predominating over the viscous ones (G′ >> G″) [13,14,53]. Interestingly, G′ and G″ values of starch-based HPP hydrogels were significantly higher than those of the commercial gel, indicative of a highly structured profile [54]. Moreover, considering the gel strength, the linear viscoelasticity range (LVR) reveals the range of deformation stress that a polymeric network withstands; thus, the wider the extension of the LVR, the higher the network strength. [47]. Remarkably, all starch-based HPP hydrogels investigated showed a wider LVR compared to Carbopol. Carbopol had already lost its gel-like behavior (G′ = G″) at 100 Pa, while in tapioca, corn, wheat, and rice starch HPP hydrogels the rheological instabilities occurred above 5000, 2000, 1000, and 300 Pa, respectively. These findings indicate the greater network strength of the latter natural structures, resulting in greater physical stability than that of commercial hydrogels. Considering the governing principles of HPP, in gels obtained utilizing this technology the physical crosslinking networks formed by the inter- and intramolecular interactions between starch and water molecules are significantly favored, and the resulting gels are characterized by strong and dense networks.

## 3. Conclusions

Starch-based HPP hydrogels are structures with high application potential in pharma, cosmetic, and food sectors. These novel structures are required to be physically stable at room temperature, and microbiological spoilage should be avoided. Thus, the determination of their stability is crucial to assess their performance with storage time. In this study, conventional and accelerated methods for the evaluation of starch-based HPP hydrogels were used. Conventional techniques demonstrated that the physical stability of starch-based HPP hydrogels depended on the occurrence of microbial spoilage and starch retrogradation, which synergically triggered the physical deterioration of the gel structures, such as network weakening and phase separation upon storage at room temperature.

Accelerated methods were proven adequate to evaluate the stability of hydrogels, requiring a shorter testing period. They allowed us to predict good physical and thermal stability and good network strength for starch-based HPP hydrogels, superior to the results for commercial hydrogels. Rheological alterations of rice, wheat, and corn HPP hydrogels were demonstrated to take place only at temperatures higher than 39 °C.

In conclusion, data obtained in this study enabled an understanding of the limits of these novel biomaterials and predictions of their physical stability, as well as improvements in their design and forecasting of their novel applications.

## 4. Materials and Methods

### 4.1. Starch-Based Hydrogel Preparation via High-Pressure Processing (HPP)

#### 4.1.1. Materials

Rice (S7260) (17.7% amylose content, 7.2% water content), wheat (S5127) (26.96% amylose content, 7.8% water content), and corn (S4126) (21.17% amylose content, 8.3% water content) starch powders were purchased from Sigma Aldrich (Steinheim, Germany). Tapioca starch (20.2% amylose content, 9.6% water content) was obtained from Rudolf Sizing Amidos do Brazil (Ibirarema—Sao Paulo, Brazil). Starch moisture content was determined according to AOAC guidelines (925.10). Amylose content was determined by an enzymatic rapid assay kit (Megazyme International Ireland Ltd., Wicklow, Ireland).

#### 4.1.2. Samples Preparation

Based on previous experimental findings [12,13], different starch–water suspensions were formed at a concentration of 20% (*w*/*w*). To ensure sample homogeneity and avoid particles sedimentation, the starch suspensions were prepared immediately before HPP treatments.

#### 4.1.3. High-Pressure Processing Treatments

HPP treatments were performed in a U22 laboratory-scale high pressure unit (Institute of High Pressure Physics, Polish Academy of Sciences, Unipress Equipment Division, Poland) as described elsewhere [55].

In each test, 10 g of the starch suspension was de-aerated, thoroughly mixed, and packed in flexible pouches, which were then sealed, loaded in the pressure vessel, and HPP-treated at 600 MPa for 15 min at 25 °C. This processing condition was sufficient for complete gelatinization and proper gel formation [14].

HPP hydrogels were stored at room temperature (20 °C) before analyses.

### 4.2. Stability Measurements

#### 4.2.1. Conventional Evaluations

Rice, wheat, corn, and tapioca HPP hydrogels were stored at 20 ± 0.2 °C in an incubator (Thermocycle, Pbi international, Milano, Italy). Microbial counts, organoleptic and texture properties, as well as swelling power and water activity levels were evaluated every four weeks. For all experimental determinations, three different samples of each starch-based hydrogel were used. Unless otherwise stated, all measurements were performed in triplicate.

##### Microbial Count

Hydrogels samples were analyzed to enumerate the numbers of mesophilic aerobic microorganisms, yeasts, and molds. Each bag containing 10 g of starch-based HPP hydrogels was aseptically opened, and together with 90 mL of buffered peptone water (VWR International, Leuven, Belgium), was introduced in an aseptic stomacher bag. A Stomacher 400 unit (Seward Laboratory, London, UK) at 260 rpm was used for the complete homogenization of the samples. Further decimal dilutions were prepared with the same diluent and plated on appropriate growth media.

To enumerate aerobic mesophilic microorganisms, 1.0 mL of each dilution was pour-plated in Plate Count Agar (PCA, Merck, Darmstadt, Germany) and incubated at 30 °C for 72 h. To count yeasts and molds, 1.0 mL of each dilution was spread-plated on Dichloran Rose Bengal Chloramphenicol medium agar (DRBC, Oxoid, Basingstoke, Hampshire, England) and then incubated for 3–5 days at 25 °C.

The microbial colonies were identified and quantified by standard methods. Microbiological data were transformed into logarithms of the number of colony-forming units (log CFU/g). The detection limit was 10 CFU/g (1.0 log CFU/g).

##### Evaluation of Organoleptic Properties

The organoleptic properties—namely the appearance, homogeneity, integrity, color, and odor—of the samples during storage were monitored using visual observations. Additionally, the physical appearances of samples were recorded using a digital camera (Sony Corp, Japan) in angular mode. Original pictures without editing and filtering were stored.

##### Swelling Properties

Swelling Power (SP)

The swelling power levels of hydrogels were determined according to the method reported by Kusumayanti et al. [56] and modified by Larrea-Wachtendorff et al. [14].

Each hydrogel sample was centrifuged (PK130R, ALC, Winchester, VA) at 1351× *g* for 10 min, the supernatant was removed, and the pellet was weighed before and after drying for 6 h at 105 °C. The swelling power was evaluated as follows:

(1) $$\mathrm{Swelling}\;\mathrm{power}\;\left(g/g\right)\;=\frac{\mathrm{Weight}\;\mathrm{of}\;\mathrm{the}\;\mathrm{wet}\;\mathrm{pellet}\;\left(g\right)}{\mathrm{Dry}\;\mathrm{weight}\;\mathrm{of}\;\mathrm{hydrogel}\;\mathrm{sample}\;\left(\mathrm{dry}\;\mathrm{basis}\right)\;\left(g\right)}$$

##### Determination of Water Activity (A_w_)

The water activity (A_w_) levels of samples were measured using a Novasina water activity instrument (TH-500, Pfäffikon, Lachen, Switzerland). Before A_w_ measurements, the instrument was calibrated at 25 °C using standard patterns. For the measurements, 1 g of hydrogel was poured into the center of the sampling chamber and consecutive readings were carried out until constant A_w_ values were attained.

##### Texture Profile Analysis (TPA)

TPA of all HPP hydrogels was performed in a TA.XT2 texture analyzer (Stable Micro Systems, Surrey, UK) equipped with a 5 kg load cell, connected to a microcomputer. The textures of hydrogel samples were determined according to the procedure reported by Larrea-Wachtendorff et al. [13]. Briefly, 6 g of hydrogel samples were poured into a cylindrical cell (24 mm height and 25 mm of internal diameter), and tests were carried out at room temperature using a P10 probe (10 mm diameter) at a rate of 1 mm/s until 50% sample deformation was attained. The compression runs were repeated twice, at a decompression rate of 1 mm/s and a delay of 5 s between two bites, to generate force–time curves. Hardness, adhesiveness, springiness, chewiness, cohesiveness, and gumminess values of hydrogels were calculated from the recorded penetration data.

#### 4.2.2. Accelerated Evaluations

##### Cycling Test

The cycling test was performed according to the method reported by Almeida and Bahia [43] with slight modifications. Hydrogels samples, obtained immediately after HPP treatments (day 0), were stored in a climatic chamber (TCN-50 plus, ARGO Lab, China) and the temperature was changed between 4 and 40 °C every 24 h for 7 days. Before undergoing TPA, all samples were stored at 20 °C for 24 h.

##### Rheological Analysis

The rheological analysis of samples was carried out in a controlled stress rheometer (AR2000-TA instruments, New Castle, DE, USA) in a plate–cone geometry configuration (40 mm diameter, 2°) with a gap of 52 µm. The viscoelastic responses of hydrogel samples were recorded (immediately obtained after HPP treatments), namely the elastic (G′) and viscous (G″) moduli under different stress (0.01 to 1000 Pa at 25 °C) and temperature (25 °C to 80 °C at 1°C/min) conditions. Additionally, for the sake of comparison, the same measurements were also carried out on commercial hydrogel samples (Diclac^®^, Eurofarma, Chile) and thermal gels. The latter were produced according to the method and processing conditions described by Larrea-Wachtendorff et al. [13].

### 4.3. Statistical Analysis

The results were analyzed using statistical descriptive analysis (mean ± SD), one-way ANOVA, and post-hoc comparison using the Fisher least significant difference (LSD) test to determine significant differences among experiments (*p*-value was <0.05). All analyses were performed using Statgraphic Centurion XVI Statistical Software (Statistical Graphics Corp., Herdon, VA, USA).

## Figures and Tables

**Table 1:** Influence of storage time on the organoleptic properties of starch-based HPP hydrogels.

HHP Hydrogel	Storage Time (Days)			
	0	30	60	90
Rice	Homogeneous Brilliant	N.M.	N.M.	More liquid
Corn	Homogeneous Opaque	N.M.	Yellowish	More liquid and yellowish
Tapioca	Homogeneous Compact	Evident Syneresis and Phase separation	Broken structure	
Wheat	Homogeneous opaque	N.M.	N.M.	Some lumps

N.M.: No modifications.

**Table 2:** Influence of temperature cycling tests on the firmness and adhesiveness of starch-based hydrogels immediately after HPP treatments.

HPP Hydrogel	Time (Day)	Firmness (N)	Adhesiveness (−N *s)
Corn	0	0.11 ± 0.01 ^c^	0.82 ± 0.11 ^a^
	7	0.06 ± 0.01 ^d^	0.36 ± 0.05 ^c^
Tapioca	0	1.36 ± 0.09 ^a^	–
	7	0.24 ± 0.07 ^b^	0.06 ± 0.00 ^e^
Rice	0	0.17 ± 0.08 ^b^	0.85 ± 0.01 ^a^
	7	0.06 ± 0.01 ^d^	0.50 ± 0.02 ^b^
Wheat	0	0.11 ± 0.01 ^c^	0.44 ± 0.05 ^bc^
	7	0.06 ± 0.00 ^d^	0.25 ± 0.02 ^d^

^a–e^ Different letters in the same column indicate significant differences among samples (LSD, *p* ≤ 0.05).

## Data Availability

The data presented in this study are available in the article.

## References

[1] Biduski B., Max W., Colussi R., Lisie S., El D.M., Lim L., Renato Á., Dias G., Zavareze R. (2018). International Journal of Biological Macromolecules Starch hydrogels: The in fl uence of the amylose content and gelatinization method. Int. J. Biol. Macromol..

[2] Mahinroosta M., Jomeh Z., Allahverdi A., Shakoori Z. (2018). Hydrogels as intelligent materials: A brief review of synthesis, properties and applications. Mater. Today Chem..

[3] McClements D.J. (2017). Recent progress in hydrogel delivery systems for improving nutraceutical bioavailability. Food Hydrocoll..

[4] Mohammadinejad R., Maleki H., Larrañeta E., Fajardo A.R., Nik A.B., Shavandi A., Sheikhi A., Ghorbanpour M., Farokhi M., Govindh P. (2019). Status and future scope of plant-based green hydrogels in biomedical engineering. Appl. Mater. Today.

[5] Ismail H., Irani M., Ahmad Z. (2013). Starch-based hydrogels: Present status and applications. Int. J. Polym. Mater. Polym. Biomater..

[6] Dong G., Mu Z., Liu D., Shang L., Zhang W., Gao Y., Zhao M., Zhang X., Chen S., Wei M. (2021). Starch phosphate carbamate hydrogel based slow-release urea formulation with good water retentivity. Int. J. Biol. Macromol..

[7] García-astrain C., Avérous L. (2018). Synthesis and evaluation of functional alginate hydrogels based on click chemistry for drug delivery applications. Carbohydr. Polym..

[8] Mun S., Kim Y.R., McClements D.J. (2015). Control of β-carotene bioaccessibility using starch-based filled hydrogels. Food Chem..

[9] Van Nieuwenhove I., Salamon A., Adam S., Dubruel P., Van Vlierberghe S., Peters K. (2017). Gelatin- and starch-based hydrogels. Part B: In vitro mesenchymal stem cell behavior on the hydrogels. Carbohydr. Polym..

[10] Qi X., Wei W., Li J., Su T., Pan X., Zuo G., Zhang J., Dong W. (2017). Design of Salecan-containing semi-IPN hydrogel for amoxicillin delivery. Mater. Sci. Eng. C.

[11] Xiao X., Yu L., Xie F., Bao X., Liu H., Ji Z., Chen L. (2017). One-step method to prepare starch-based superabsorbent polymer for slow release of fertilizer. Chem. Eng. J..

[12] Larrea-Wachtendorff D., Di Nobile G., Ferrari G. (2020). Effects of processing conditions and glycerol concentration on rheological and texture properties of starch-based hydrogels produced by high pressure processing (HPP). Int. J. Biol. Macromol..

[13] Larrea-Wachtendorff D., Tabilo-Munizaga G., Ferrari G. (2019). Potato starch hydrogels produced by high hydrostatic pressure (HHP): A first approach. Polymers.

[14] Larrea-Wachtendorff D., Sousa I., Ferrari G. (2020). Starch-Based Hydrogels Produced by High-Pressure Processing (HPP): Effect of the Starch Source and Processing Time. Food Eng. Rev..

[15] Barba F.J., Terefe N.S., Buckow R., Knorr D., Orlien V. (2015). New opportunities and perspectives of high pressure treatment to improve health and safety attributes of foods. A review. Food Res. Int..

[16] Knorr D., Heinz V., Buckow R. (2006). High pressure application for food biopolymers. Biochim. Biophys. Acta-Proteins Proteom..

[17] Stute R., Klingler R.W., Boguslawski S., Eshtiaghi M.N., Knorr D. (1996). Effects of high pressures treatment on starches. Starch/Starke.

[18] Błaszczak W., Fornal J., Kiseleva V.I., Yuryev V.P., Sergeev A.I., Sadowska J. (2007). Effect of high pressure on thermal, structural and osmotic properties of waxy maize and Hylon VII starch blends. Carbohydr. Polym..

[19] Błaszczak W., Valverde S., Fornal J. (2005). Effect of high pressure on the structure of potato starch. Carbohydr. Polym..

[20] Błaszczak W., Buciński A., Górecki A.R. (2015). In vitro release of theophylline from starch-based matrices prepared via high hydrostatic pressure treatment and autoclaving. Carbohydr. Polym..

[21] Buckow R., Heinz V., Knorr D. (2007). High pressure phase transition kinetics of maize starch. J. Food Eng..

[22] Katopo H., Song Y., Jane J.L. (2002). Effect and mechanism of ultrahigh hydrostatic pressure on the structure and properties of starches. Carbohydr. Polym..

[23] Kawai K., Fukami K., Yamamoto K. (2012). Effect of temperature on gelatinization and retrogradation in high hydrostatic pressure treatment of potato starch—water mixtures. Carbohydr. Polym..

[24] Li W., Tian X., Liu L., Wang P., Wu G., Zheng J., Ouyang S., Luo Q., Zhang G. (2015). High pressure induced gelatinization of red adzuki bean starch and its effects on starch physicochemical and structural properties. Food Hydrocoll..

[25] Li W., Bai Y., Mousaa S.A.S., Zhang Q., Shen Q. (2012). Effect of High Hydrostatic Pressure on Physicochemical and Structural Properties of Rice Starch. Food Bioprocess Technol..

[26] Bajaj S., Singla D., Sakhuja N. (2012). Stability testing of pharmaceutical products. J. Appl. Pharm. Sci..

[27] Reyes J.E., Guanoquiza M.I., Tabilo-Munizaga G., Vega-Galvez A., Miranda M., Pérez-Won M. (2012). Microbiological stabilization of Aloe vera (*Aloe barbadensis* Miller) gel by high hydrostatic pressure treatment. Int. J. Food Microbiol..

[28] Daryaei H., Yousef A.E., Balasubramaniam V.M. (2016). Microbiological aspects of high pressure food processing: Inactivation of vegetative microorganisms and spores. High Pressure Processing of Food.

[29] Reyes J.E., Tabilo-Munizaga G., Pérez-Won M., Maluenda D., Roco T. (2015). Effect of high hydrostatic pressure (HHP) treatments on microbiological shelf-life of chilled *Chilean jack* mackerel (*Trachurus murphyi*). Innov. Food Sci. Emerg. Technol..

[30] Modugno C., Peltier C., Simonin H., Dujourdy L., Capitani F., Sandt C., Perrier-Cornet J.M. (2020). Understanding the Effects of High Pressure on Bacterial Spores Using Synchrotron Infrared Spectroscopy. Front. Microbiol..

[31] Nguyen Thi Minh H., Dantigny P., Perrier-Cornet J.M., Gervais P. (2010). Germination and inactivation of Bacillus subtilis spores induced by moderate hydrostatic pressure. Biotechnol. Bioeng..

[32] Kawai K., Fukami K., Yamamoto K. (2007). State diagram of potato starch-water mixtures treated with high hydrostatic pressure. Carbohydr. Polym..

[33] Kawai K., Fukami K., Yamamoto K. (2007). Effects of treatment pressure, holding time, and starch content on gelatinization and retrogradation properties of potato starch-water mixtures treated with high hydrostatic pressure. Carbohydr. Polym..

[34] Chung Y.L., Lai H.M. (2004). Water status of two gelatin gels during storage as determined by magnetic resonance imaging. J. Food Drug Anal..

[35] Torres M.D., Fradinho P., Raymundo A., Sousa I., Falqué E., Domínguez H. (2021). The key role of thermal waters in the development of innovative gelled starch-based matrices. Food Hydrocoll..

[36] BeMiller J.N., Whistler R. (2009). Starch: Chemistry and Technology.

[37] Wang S., Li C., Copeland L., Niu Q., Wang S. (2015). Starch Retrogradation: A Comprehensive Review. Compr. Rev. Food Sci. Food Saf..

[38] BeMiller J.N. (2018). Carbohydrate Chemistry for Food Scientists.

[39] Scott W.J. (1957). Water Relations of Food Spoilage Microorganisms. Adv. Food Res..

[40] Pycia K., Gałkowska D., Juszczak L., Fortuna T., Witczak T. (2015). Physicochemical, thermal and rheological properties of starches isolated from malting barley varieties. J. Food Sci. Technol..

[41] Wang P., Wang P., Tian J., Yu X., Chang M., Chu X., Wu N. (2016). A new strategy to express the extracellular α-amylase from Pyrococcus furiosus in Bacillus amyloliquefaciens. Sci. Rep..

[42] Kommanaboyina B., Rhodes C.T. (1999). Trends in stability testing, with emphasis on stability during distribution and storage. Drug Dev. Ind. Pharm..

[43] Almeida I.F., Bahia M.F. (2006). Evaluation of the physical stability of two oleogels. Int. J. Pharm..

[44] Carstensen J.T., Rhodes C.T. (1986). Cyclic testing in stability programs. Drug Dev. Ind. Pharm..

[45] Schirmer M., Jekle M., Becker T. (2015). Starch gelatinization and its complexity for analysis. Starch/Starke.

[46] Taherian A.R., Fustier P., Ramaswamy H.S. (2008). Steady and dynamic shear rheological properties, and stability of non-flocculated and flocculated beverage cloud emulsions. Int. J. Food Prop..

[47] Abdul Rahman M.N., Qader O.A.J.A., Sukmasari S., Ismail A.F., Doolaanea A.A. (2017). Rheological characterization of different gelling polymers for dental gel formulation. J. Pharm. Sci. Res..

[48] Doona C.J., Feeherry F.E., Baik M.Y. (2006). Water dynamics and retrogradation of ultrahigh pressurized wheat starch. J. Agric. Food Chem..

[49] Hu X., Xu X., Jin Z., Tian Y., Bai Y., Xie Z. (2011). Retrogradation properties of rice starch gelatinized by heat and high hydrostatic pressure (HHP). J. Food Eng..

[50] Yang Z., Chaib S., Gu Q., Hemar Y. (2017). Food Hydrocolloids Impact of pressure on physicochemical properties of starch dispersions. Food Hydrocoll..

[51] Biliaderis C.G. (2009). Structural Transitions and Related Physical Properties of Starch. Starch.

[52] Fang F., Tuncil Y.E., Luo X., Tong X., Hamaker B.R., Campanella O.H. (2019). Shear-thickening behavior of gelatinized waxy starch dispersions promoted by the starch molecular characteristics. Int. J. Biol. Macromol..

[53] Torres M.D., Chenlo F., Moreira R. (2018). Rheological Effect of Gelatinisation Using Different Temperature-Time Conditions on Potato Starch Dispersions: Mechanical Characterisation of the Obtained Gels. Food Bioprocess Technol..

[54] Lapasin R., Matricardi P., Alhaique F., Coviello T. (2016). Rheological Characterization of Hydrogels. Polysaccharide Hydrogels.

[55] De Maria S., Ferrari G., Maresca P. (2017). Effect of high hydrostatic pressure on the enzymatic hydrolysis of bovine serum albumin. J. Sci. Food Agric..

[56] Kusumayanti H., Handayani N.A., Santosa H. (2015). Swelling Power and Water Solubility of Cassava and Sweet Potatoes Flour. Procedia Environ. Sci..

